# Interventricular Membranous Septal Aneurysm in an Asymptomatic Patient

**DOI:** 10.1016/j.case.2025.11.002

**Published:** 2025-12-22

**Authors:** Daniel J. Lim, Felix M. Uy, Wei L. Huang

**Affiliations:** Department of Cardiology, Changi General Hospital, Singapore

**Keywords:** Interventricular membranous septal aneurysm, Multimodality cardiac imaging, Computed tomography, Echocardiography

## Abstract

• Incidental IVMSA is found on CCT and TTE.
• Coronal CCT images improved precise anatomical localization of IVMSA.
• Multimodal imaging is necessary to confirm the diagnosis of an IVMSA.
• Multimodal imaging helps guide management and exclude complications.

## Graphical abstract

**Figure:**
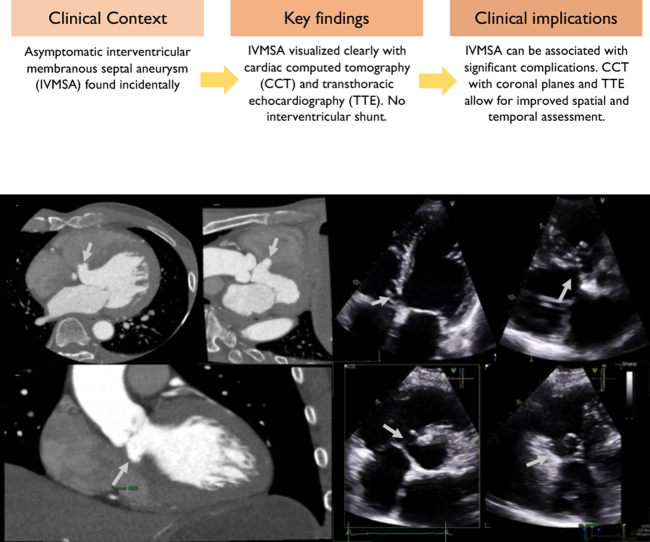

## Introduction

Interventricular membranous septal aneurysm (IVMSA) formation is a rare condition, often detected incidentally during coronary angiography or multimodality cardiac imaging and frequently associated with ventricular septal defect (VSD) or other congenital heart disease.^1^^,^^2^ The membranous septum is the basal fibrous portion of the interventricular septum, immediately inferior to the aortic annulus and adjacent to the atrioventricular (AV) conduction axis.^2^ As this region lacks myocardial tissue, high-pressure gradients may contribute to the development of an IVMSA.^1^ The etiologies proposed include idiopathic, traumatic, infective, or spontaneous VSD closure. Recognition remains important despite its rarity, as even infrequent complications such as conduction disturbances, right ventricular (RV) outflow tract (RVOT) obstruction, bacterial endocarditis, and stroke can have significant clinical consequences.^2^, ^3^, ^4^, ^5^ Given the relative obscurity of this condition, this case report aims to present a classic example of an IVMSA seen on transthoracic echocardiography (TTE) and cardiac computed tomography (CCT) that was an incidental finding in an asymptomatic patient.

## Case Presentation

A 55-year-old man with a history of hepatitis B cirrhosis on entecavir and no cardiac history was admitted for atypical chest pain, which was described as a left upper chest pain that was sharp in nature, worse with lifting of the arm. and present for many months with no improvement. It was associated with exertional breathlessness relieved by rest. The physical examination was largely unremarkable, with chest pain that was reproducible on palpation, no murmurs, and clear breath sounds bilaterally. There was no significant rise in high-sensitivity cardiac troponins, and electrocardiogram (ECG) showed a prolonged PR interval of 230 ms and sinus bradycardia of 37 to 51 beats per minute with no dynamic ST-T segment changes.

**Figure 1:**
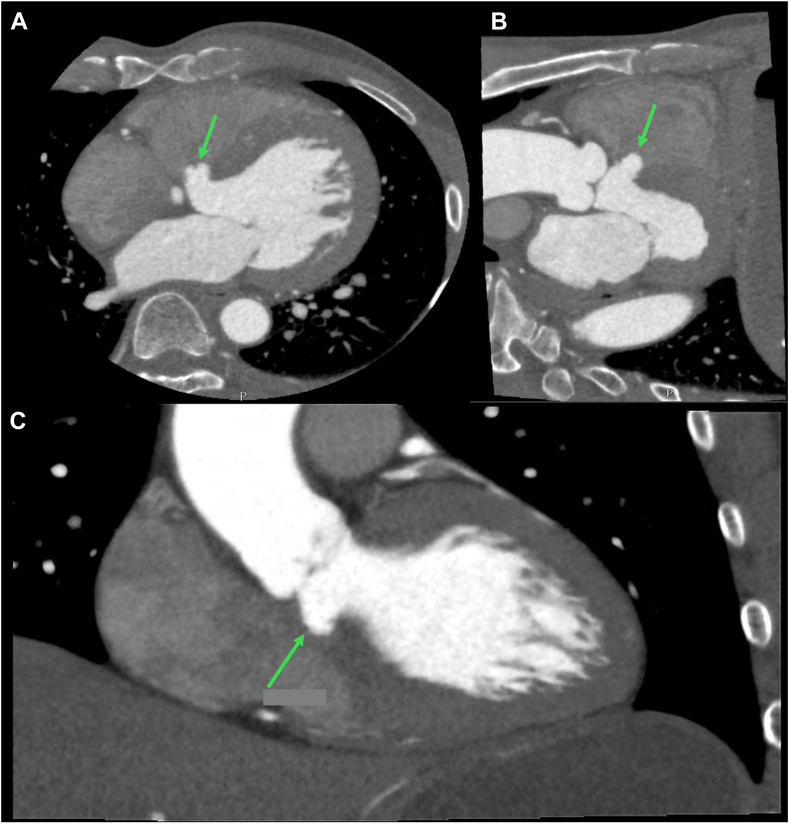
Cardiac computed tomography, multiplanar reconstruction (0.5 mm slice; 75% R-R), oblique axial **(A)**, sagittal **(B)** and coronal **(C)**, demonstrates normal cardiac dimensions and a saccular outpouching arising from the membranous septum with a narrow neck adjacent to the aortic annulus and tricuspid septal leaflet projecting toward the right ventricle, consistent with an IVMSA (*arrows*).

The patient underwent a CCT for evaluation of coronary arteries, and a TTE was done to evaluate dyspnea on exertion. The CCT showed moderate stenosis in the distal left main and ostial left anterior descending artery and minimal to mild stenoses elsewhere in the coronary arteries with a coronary artery calcium score of 174 (Agatston), which is between the 50th and 75th percentiles matched for age and gender. Shallow myocardial bridging of the mid left anterior descending artery with no significant stenosis was present. A defect in the basal ventricular septum was initially reported on the CCT, which was subsequently better visualized with coronal plane reconstructions, localizing it to being just inferior to the aortic annulus, with normal apposition of aortic valve leaflets during diastole. Contrast agent did not enter the RV cavity. This was suggestive of a membranous ventricular septal aneurysm that did not appear to extend into the RVOT (Figure 1).

**Figure 2:**
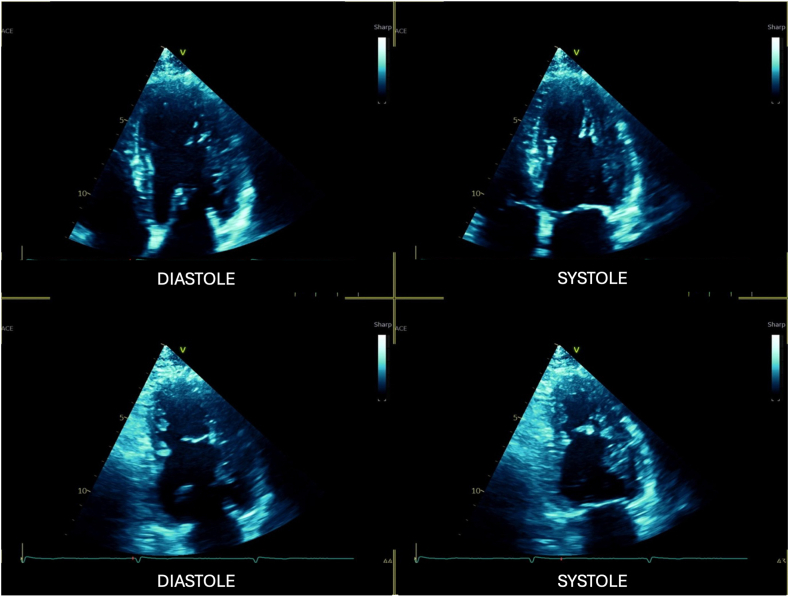
Two-dimensional TTE, apical 4-chamber (*top*) and apical 2-chamber (*bottom*) views in diastole (*left*) and systole (*right*), demonstrates normal LV size with moderately reduced LV global systolic function (LVEF = 37%) without regional wall motion abnormalities; the IVMSA is not visualized in these conventional imaging planes.

**Figure 3:**
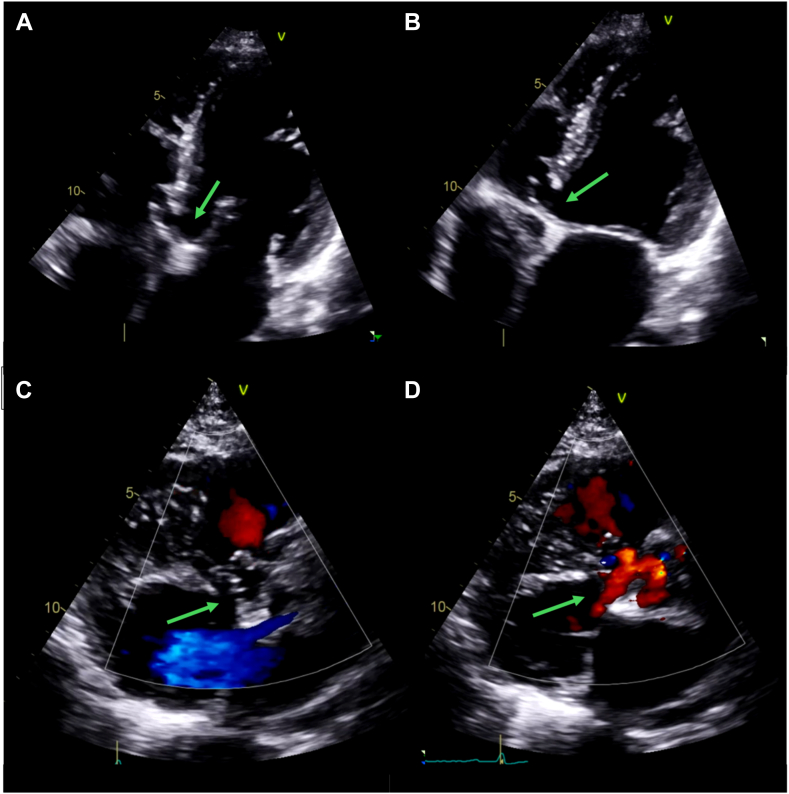
Two-dimensional TTE without (*top*) and with (*bottom*) color-flow Doppler, modified apical 4-chamber **(A** and **B**) and parasternal long-axis **(C** and **D**), diastolic **(A, C)**, and systolic **(B, D)** views, demonstrates a narrow-necked saccular outpouching anatomic defect that arises from the membranous septum, extends into the right ventricle adjacent to the septal tricuspid leaflet, is noncontracting, and is without evidence of left-to-right shunt, consistent with an IVMSA (*arrows*).

**Figure 4:**
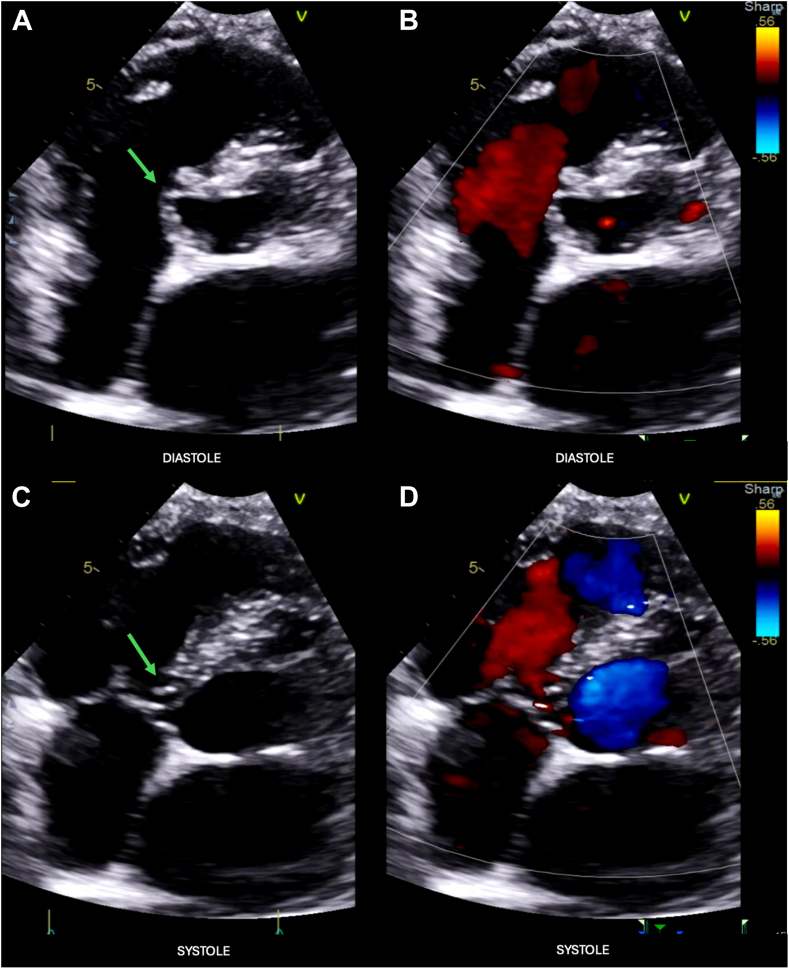
Two-dimensional TTE without (*left*) and with (*right*) color-flow Doppler, basal parasternal long-axis diastolic **(A)** and **B** and systolic **(C** and **D**) views, demonstrates a narrow-necked saccular outpouching anatomic defect that arises from the membranous septum, just beneath the aortic annulus, extends into the right ventricle adjacent to the septal tricuspid leaflet, is noncontracting, and is without evidence of left-to-right shunt, consistent with an IVMSA (*arrows*).

The TTE showed a non-dilated left ventricle with moderately reduced systolic function, with a left ventricular (LV) ejection fraction (LVEF) of 37% (Simpson's biplane) with moderate global hypokinesia without discrete regional wall motion abnormalities of the left ventricle (Figure 2, Video 2). A saccular dilatation of the basal interventricular septum was seen that suggested an aneurysm without evidence of an associated shunt (Figures 3 and 4; Videos 1, 3 and 4). The aneurysm was off axis relative to the apical planes and therefore not included in the contours for the measurement of LVEF. Other TTE findings were normal RV size and function and normal aortic valve function.

In view of the left main coronary disease, the patient was started on single antiplatelet therapy and scheduled for an outpatient discussion on invasive coronary angiography. A 24-hour ambulatory ECG monitor was also planned to assess sinus bradycardia. Guideline-directed therapy for heart failure with reduced ejection fraction was initiated, and the patient remained symptom-free during hospitalization. Subsequently, a diagnostic coronary angiogram was performed that showed minor coronary artery disease and the patient was scheduled for cardiovascular magnetic resonance (CMR) and follow-up with the adult congenital heart disease specialists.

## Discussion

The membranous portion of the septum is a small segment situated beneath the attachment points of the right and non-coronary cusps of the aortic valve, extending to both the inlet and outlet regions of the muscular septum. The interventricular membranous septum is generally an oval or triangular area, around 50 mm^2^ in size, composed of the endocardial lining of the heart chambers and supported solely by dense fibrous tissue.^2^^,^^6^ Interventricular membranous septal aneurysm is a common incidental finding in CCT that is used for aortic valve surgery planning.^1^^,^^7^ This case reports improved visualization of the IVMSA upon the addition of coronal cuts to the CCT, which was corroborated by Carcano *et al.*^2^ In addition to CCT, CMR can offer detailed imaging of the septum using T1/T2-weighted double-inversion recovery-prepared black blood sequences for morphology and balanced steady-state free precession cine images to demonstrate intermittent bulging. Beyond cine and flow sequences for septal defects, CMR can also evaluate for thrombus within the IVMSA using long inversion time late gadolinium enhancement to suppress blood signal, with early-phase imaging (i.e., early gadolinium enhancement/microvascular obstruction) demonstrating persistent nonenhancement consistent with thrombus.^2^ No thrombus was identified in our patient.

A basal IVMSA can distort the LV contour near the annulus, resulting in an underestimation of the LVEF with TTE two-dimensional Simpson's biplane. However, in our patient, the IVMSA remained off axis and excluded from the apical 4-chamber/apical 2-chamber endocardial contours; thus the Simpson's biplane estimation of LVEF remained robust. Where results would impact care, it would be judicious to confirm LVEF measurement with CMR or three-dimensional echocardiography, which are less geometry dependent. Single-photon emission computed tomography or positron emission tomography is helpful to determine whether the reduced LVEF is related to coronary ischemia. In our case, there was global hypokinesia with minor coronary artery disease, no shunt, and no RVOT impingement, warranting the planned CMR to evaluate for nonischemic causes.

The diagnosis in this case was established by the specific anatomic details that included the location below the aortic annulus on the LV side, the lack of contrast with CCT or color Doppler flow entering the right ventricle with TTE, and no findings of a characteristic “windsock” protrusion into the right ventricle.^8^ The absence of visualized synchronous contraction of the surrounding myocardium suggested this was not an LV diverticulum.^2^ A sinus of Valsalva aneurysm would be located above the aortic annulus and is commonly associated with aortic regurgitation.^2^ A congenital muscular septal aneurysm is considered unlikely given it typically arises from the muscular rather than membranous portion of the interventricular septum and displays an age-dependent morphologic evolution.^9^ Furthermore, the CMR confirmed that it was not a basal septal, post-infarct aneurysm by demonstrating a lack of the characteristic endocardial pattern of late gadolinium enhancement.^10^

The clinical presentation and course of the IVMSA relates to the specific morphology of the bulging and presence and association of defects, shunts, and complications. While our patient was asymptomatic, previous cases have reported symptoms including fatigue, exertional dyspnea from severe RV dysfunction, or cyanosis from right-to-left shunting across a VSD in the aneurysm.^3^^,^^11^ A cardiac murmur and thrill may be present, attributed to the presence of the aneurysmal sac bulging into the RVOT, typically indistinguishable between that of patients with VSDs.^11^ While most present with no hemodynamic instability, occasionally there can be ECG findings of right-axis deviation, RV hypertrophy, and right atrial (RA) enlargement.^2^ This occurs when IVMSAs protrude into the RVOT, causing chronic pressure overload and progressive RV remodeling.^3^^,^^11^ In cases with a residual perimembranous VSD, left-to-right shunting results in RV volume loading, with associated RA enlargement; if pulmonary pressures rise, RV hypertrophy may ensue, shifting the ECG axis rightward.^3^ Further progression of this aneurysmal bulging can subsequently lead to RVOT obstruction.^3^^,^^11^ In our patient, no shunt or RVOT obstruction was present; thus the ECG did not show any RA enlargement, RV hypertrophy, or RA deviation.

Aortic regurgitation, a potential complication of IVMSAs, can be visualized with left coronal oblique and left sagittal oblique planes on CCT with further corroboration on echocardiographic imaging.^12^ Transesophageal echocardiography can thereafter be used to characterize the IVMSA, allowing for visualization of real-time movement and diagnostic certainty of interventricular flow to plan surgical repair.^13^

Arrhythmias and complete AV block are not uncommon given the membranous septum's location in an important electrical area of the heart.^2^ The AV node is located at the base of the atrial septum within Koch's triangle, the apex of which is the AV part of the membranous septum. The AV bundle fibers run along its posterior and inferior margins,^14^ and stretching or surgical manipulation of the aneurysmal base may threaten adjacent conduction tissue, with AV block postulated as a possible complication.^15^ In our case, given there was an asymptomatic prolonging of the PR interval to 230 ms, it warranted close follow-up and swift action in the event of any progression to high-grade AV blocks. However, given the site of compression by the IVMSA is often at the AV node, it is unlikely that the sinus bradycardia witnessed in our patient was a byproduct of the IVMSA.

Thromboembolism and stroke have been reported in cases, possibly predisposed by the turbulence of blood flow along the IVMSA.^4^^,^^5^ Blood stasis within the sac has been linked to embolic stroke, highlighting the importance of meticulous thrombus surveillance on multimodality imaging and consideration of anticoagulation when thrombus is present.^5^

Lastly, IVMSA may predispose the individual to bacterial endocarditis due to the high-velocity flow of blood passing along the aneurysm, allowing platelet and fibrin adhesion to the exposed endothelium and forming a sterile thrombus with the potential to harbor microorganisms.^4^

The wide spectrum of clinically significant complications associated with IVMSAs, despite it commonly being an incidental finding, have warranted the recommendation of periodic echocardiography.^2^ Surgical intervention is rarely needed, and suggested treatments are often tailored to the complications that develop.^5^

## Conclusion

This case underscores the importance of recognizing IVMSA as an anatomic variant with potential clinical implications despite its usual hemodynamic quiescence. Its proximity to the cardiac conduction system and aortic valve highlights the need for careful differentiation from other septal pathologies. Multimodality imaging, particularly TTE, CCT, and CMR, plays a central role in establishing diagnosis, defining anatomy, and excluding complications. Awareness of this entity allows for appropriate surveillance and prevention of misdiagnosis or unnecessary intervention.

## Ethics Statement

The authors declare that the work described has been carried out in accordance with The Code of Ethics of the World Medical Association (Declaration of Helsinki) for experiments involving humans.

## Consent Statement

Complete written informed consent was obtained from the patient (or appropriate parent, guardian, or power of attorney) for the publication of this study and accompanying images.

## Funding

The authors declare that this report did not receive any specific grant from funding agencies in the public, commercial, or not-for-profit sectors.

## Data Statement

The data that support the findings of this study are available from the corresponding author upon reasonable request.

## Disclosure Statement

The authors reported no actual or potential conflicts of interest relative to this document.

## Supplementary Data

Video 1 Two-dimensional TTE, modified apical 4-chamber view, demonstrates normal LV size with systolic dysfunction and a narrow-necked saccular outpouching anatomic defect that arises from the membranous septum, extends into the right ventricle adjacent to the septal tricuspid leaflet, and is noncontracting, consistent with an IVMSA.

Video 2 Two-dimensional TTE, apical 4-chamber view, demonstrates normal LV size with moderately reduced LV global systolic function (LVEF = 37%) without regional wall motion abnormalities; the IVMSA is not visualized in conventional imaging planes.

Video 3 Two-dimensional TTE, modified parasternal long-axis view with color-flow Doppler, demonstrates a narrow-necked saccular outpouching anatomic defect that arises from the membranous septum, extends into the right ventricle adjacent to the septal tricuspid leaflet, is noncontracting, and is without evidence of left-to-right shunt, consistent with an IVMSA.

Video 4 Two-dimensional TTE, without (*left*) and with (*right*) color-flow Doppler, basal parasternal long-axis views, demonstrates a narrow-necked saccular outpouching anatomic defect that arises from the membranous septum just beneath the aortic annulus, extends into the right ventricle adjacent to the septal tricuspid leaflet, is noncontracting, and is without evidence of left-to-right shunt, consistent with an IVMSA.
